# p32 is Required for Appropriate Interleukin-6 Production Upon LPS Stimulation and Protects Mice from Endotoxin Shock

**DOI:** 10.1016/j.ebiom.2017.05.018

**Published:** 2017-05-11

**Authors:** Katsuhiko Sasaki, Kazuhito Gotoh, Sho Miake, Daiki Setoyama, Mikako Yagi, Ko Igami, Takeshi Uchiumi, Donchon Kang

**Affiliations:** a Department of Clinical Chemistry and Laboratory Medicine, Graduate School of Medical Sciences, Kyushu University, 3-1-1, Maidashi, Higashi-ku, Fukuoka 812-8582, Japan; b Medical Solution Promotion Department, Business Management Center, Medical Solution Segment, LSI Medience Corporation, 4-1, Kyudaishimmachi, Nishi-ku, Fukuoka 819-0388, Japan

**Keywords:** LPS, Il-6, Mitochondria, ATF4, p32

## Abstract

Sepsis is a major cause of morbidity and mortality in seriously ill patients and mitochondrial dysfunction is associated with poor outcomes in septic patients. Although interleukin-6 (IL-6) is a good prognostic marker for sepsis, the relationship between mitochondrial dysfunction and IL-6 remains poorly understood. We identified p32/C1QBP/HABP1 as a regulator of IL-6 production in response to lipopolysaccharide (LPS). LPS induced IL-6 overproduction in p32 deficient mouse embryonic fibroblasts (MEFs) through NF-κB independent but activating transcription factor (ATF) 4 dependent pathways. Short hairpin RNA-based knockdown of ATF4 in p32 deficient MEFs markedly inhibited LPS-induced IL-6 production. Furthermore, MEFs treated with chloramphenicol, an inhibitor of mitochondrial translation, produced excessive IL-6 via ATF4 pathways. Using a LPS-induced endotoxin shock model, mice with p32 ablation in myeloid cells showed increased lethality and overproduction of IL-6. Thus, this study provides a molecular link how mitochondrial dysfunction leads to IL-6 overproduction and poor prognosis of sepsis.

## Highlights

• Loss of p32 increases LPS-induced IL-6 production via the ATF4 activation pathway.
• Inhibition of mitochondrial translation increases LPS-induced IL-6 overproduction.
• p32 protects mice from endotoxin shock.

Sepsis-associated multiple organ dysfunction syndrome (MODS) still carries a significant morbidity and mortality. Mitochondrial dysfunction is associated with poor outcomes in patients with sepsis and MODS. IL-6 has been used as a prognostic marker for outcomes in severe septic patients, but it is still unknown whether sepsis-induced mitochondria dysfunction is associated with the production of IL-6. This study demonstrates that p32/C1QBP/HABP1, which is essential for mitochondrial translation, is a regulator of IL-6 production upon LPS stimulation and protects mice from endotoxin shock. Therefore, p32 could be a therapeutic target for controlling LPS-related inflammatory diseases in humans.

## Graphical abstract

**Figure:**
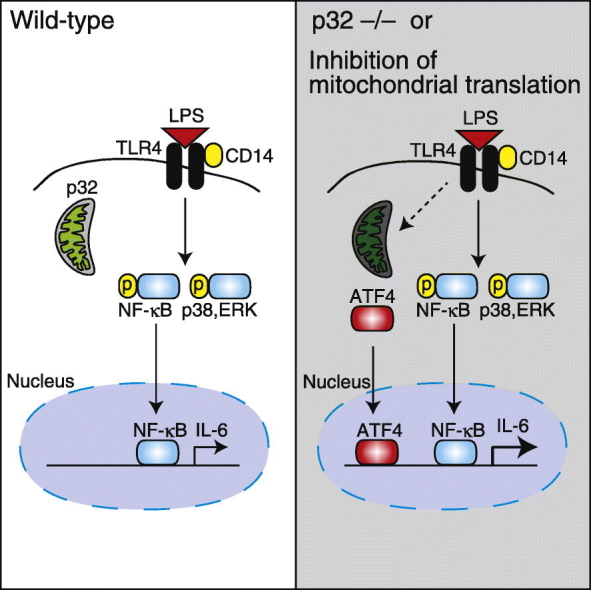

## 1 Introduction

Sepsis is a life-threatening condition that results from a harmful host response to infection. Sepsis-associated multiple organ dysfunction syndrome (MODS) is the predominant cause of mortality among seriously ill patients (Deitch, 1992). Despite improvements in laboratory testing and clinical therapy, the mortality rate of severe sepsis remains at approximately 30% and hospital admissions for severe sepsis have increased (Opal et al., 2013) (Caironi et al., 2014) (Lagu et al., 2012). Because around 750,000 patients are diagnosed with severe sepsis in the United States each year (Angus et al., 2001), improvements in care for severe sepsis remain a priority.

Mitochondria regulate many aspects of cellular signaling and metabolic pathways through the fatty acid oxidation (FAO), the tricarboxylic acid cycle and the electron transport chain. Several lines of evidence suggest that mitochondrial dysfunction is associated with poor outcomes in patients with sepsis and MODS (Brealey et al., 2002) (Brealey et al., 2004) (Duvigneau et al., 2008). While the causality is not yet confirmed, it does nevertheless suggest a new route for therapeutic intervention focused on either mitochondrial protection or acceleration of the recovery process during sepsis (Singer, 2014).

Endotoxin/lipopolysaccharide (LPS), a component of the outer membrane of Gram-negative bacteria, has been widely used in sepsis research. Expressed at the cell surface, toll like receptor (TLR) 4 detects LPS from Gram-negative bacteria. Interaction of TLR4 with LPS induces several intracellular signaling molecules, leading to the expression of nuclear factor-κB (NF-κB)-dependent pro-inflammatory cytokines or interferon regulatory factor-dependent type I interferons (IFNs) (Kawai and Akira, 2010). The inflammatory response is orchestrated by proinflammatory cytokines such as tumor necrosis factor (TNF), interleukin (IL)-1, and IL-6.

IL-6 is a pleiotropic cytokine that plays a major role in host defense by regulating immune and inflammatory responses. During infection and chronic inflammation, IL-6 is produced by various types of cells, such as fibroblasts, macrophages, dendritic cells, T-cells, B-cells, keratinocytes, endothelial cells, mesangial cells, adipocytes and some tumor cells. Importantly, fibroblasts like other immune cells are a major source of IL-6. In chronic inflammation, fibroblasts are recognized as important regulators of inflammation through the production of IL-6 (Bernardo and Fibbe, 2013). In spite of the presence of several negative feedback mechanisms, constitutive overproduction of IL-6 is responsible for the pathogenesis of various inflammatory diseases, such as rheumatoid arthritis, systemic juvenile arthritis, and Crohn's disease (Kishimoto, 2010). Therefore, IL-6 signaling blockade with the humanized anti-IL-6 receptor antibody, tocilizumab, is a novel therapeutic strategy for various autoimmune and inflammatory diseases (Kishimoto, 2010).

Moreover, several studies have investigated the validity of using IL-6 for early sepsis diagnosis and predicting mortality (Viallon et al., 2000) (Gardlund et al., 1995) (Pettila et al., 2002). IL-6 has also been used as a prognostic marker for outcomes in septic patients, but it is still unknown whether sepsis-induced mitochondria dysfunction is associated with the production of IL-6.

Human p32/C1QBP/HABP has been cloned as a splicing factor 2-associated protein from human HeLa cells (Krainer et al., 1991). The p32 protein is a doughnut-shaped trimer, which primarily localizes to the mitochondrial matrix, but has also been reported to be present at the cell surface, in the nucleus and cytosol, as well as within secretory granules (Jiang et al., 1999) (Matthews and Russell, 1998) (van Leeuwen and O'Hare, 2001) (Soltys et al., 2000). We previously showed that p32 is synthesized as a pre-protein and is processed by proteolytic cleavage of the N-terminal amino acids containing the mitochondrial signal sequence (Muta et al., 1997). In addition, we reported that mitochondrial p32 is essential for mouse embryonic development dependent on mitochondrial translation (Yagi et al., 2012). Several previous siRNA studies of innate immunity suggest that p32 suppresses antiviral immune responses (Xu et al., 2009b) (Wang et al., 2014b) (Liu et al., 2015), but its molecular mechanisms are little elucidated. Much less is known about the role of p32 in antibacterial immunity.

Mitochondrial functions, such as mitochondrial membrane potential and mitochondrial reactive oxygen species (mROS), play important roles in innate antiviral immunity (Koshiba et al., 2011) (Tal et al., 2009) (Koshiba, 2013). On the other hand, many relationships between mitochondrial functions and sepsis remain to be revealed. In this paper, we present evidence that p32 is critically involved in TLR4-mediated IL-6 induction in vitro and in vivo. We have found that loss of mitochondrial translation due to a deficiency of p32 or the presence of inhibitors increases LPS-induced IL-6 overproduction via ATF4 activation in mouse embryonic fibroblasts (MEFs). Additionally, using a mice endotoxin shock model, we showed that p32 protects mice from endotoxin shock.

## 2 Material and Methods

### 2.1 Cell Preparation and Retrovirus–mediated Gene Transfer

MEFs were isolated from wild-type and p32 knockout mice as described previously (Yagi et al., 2012). MEFs were maintained at 37 °C and 5% CO2 in Dulbecco's modified Eagle's medium (DMEM; Sigma-Aldrich, USA) supplemented with 10% FBS. To generate bone marrow-derived macrophages (BMMQs), bone marrow cells were cultured for 7 days with macrophage colony-stimulating factor (20 ng/mL; PeproTech, USA).

pSUPER retro puro GFP shRNA was a gift from John Gurdon (Addgene plasmid # 30519). The retroviral vector pSUPER retro puro were used to generate the plasmid encoding *GFP* shRNA or *Atf4* shRNA. The shRNA target sequences were: Mouse ATF4: 5′-AAGAGAAGGCAGATTCTCT-3′. The retroviral vector pMX was used to generate the plasmid encoding IRES–GFP (Empty), or *p32*–IRES–GFP. Retroviral transduction was done as previously described (Gotoh et al., 2010). These plasmids were transfected into Platinum-E packaging cells using FuGENE 6 transfection reagent (Roche Applied Science, Germany). The cell-culture supernatants were harvested 48 h after transfection, supplemented with polybrene (5 μg/mL; Sigma-Aldrich, USA), and used to infect MEFs and BMMQs. Before assay, two additional retroviral infections were performed at daily intervals.

### 2.2 Reagents

Actinomycin D, LPS, Rotenone, Antimycin, Oligomycin, Etomoxir and antibodies against β-actin were purchased from Sigma-Aldrich, USA, and Chloramphenicol were obtained from Wako Pure Chemicals, Japan. Actinonin was purchased from Enzo Life Sciences, Germany. Polyclonal antibodies against mouse p32 were raised in our laboratory. Antibodies against p38, phospho-p38, Erk1/2, phospho-Erk1/2, NFκB p65, phospho-NFκB p65, IκB, phospho-IκB, ATF4 were purchased from Cell Signaling Technology, USA. Anti-COX1 antibodies and anti-B23 antibodies were from Thermo Fisher scientific, USA. Total OHPHOS rodent antibodies cocktail were from Abcam, USA.

### 2.3 Real-time PCR

After treatment with RNase-free DNase I (QIAGEN, Germany), RNA samples were reverse transcribed PrimeScript™ RT Reagent Kit (TAKARA, Japan) according to the manufacturer's instructions. Quantitative real-time RT-PCR analysis was performed using specific primers (Supplementary Table S1). The expression of the genes was detected by qPCR with a thermal cycler (StepOne plus; Applied Biosystems).

MEFs were stimulated with LPS (100 ng/mL) for 3 h. Actinomycin D (10 μg/mL) was then added to cell culture medium to stop transcription. After incubated with actinomycin D, total RNAs were prepared at the indicated time periods.

### 2.4 Immunoblotting Analysis and Nuclear extraction

For direct immunoblotting, MEFs and macrophages were lysed with cell lysis buffer (50 mM Tris–HCl, pH 7.5, 1 mM EDTA, 150 mM NaCl and 0.5% NP-40) and then subjected to immunoblotting using specific antibodies.

For nuclear extraction, MEFs were washed with cold Phosphate buffered saline (PBS). The cells were suspended in Hypotonic Buffer (10 mM HEPES (pH 7.9), 1.5 mM MgCl2, 10 mM KCl, 1 mM Dithiothreitol, and protease inhibitor cocktail). After incubation on ice for 15 min, the cells were added to the 0.6% IGEPAL CA-630 solution and vortexed vigorously for 10 s. Next, the cells were centrifuged at 10,000*g* for 30 s, and the supernatants were transferred to a fresh tube as cytosol fraction. The crude nuclei pellet was resuspended in extraction buffer (20 mM HEPES, pH 7.9, with 1.5 mM MgCl2, 0.42 M NaCl, 0.2 mM EDTA, 25% (v/v) Glycerol, 1 mM Dithiothreitol, and protease inhibitor cocktail), with intermittent vortexing for 15 min. Finally, cells were centrifuged for 5 min at 14,000*g* and the supernatant was transferred to a fresh tube as nuclear fraction.

### 2.5 Immunofluorescence Microscopy

After stimulation with LPS (100 ng/mL) at the indicated time periods, the cells were fixed with 4% paraformaldehyde/PBS for 10 min and permeabilized with 0.2% TritonX-100/PBS for 5 min. After being blocked with 1% bovine serum albumin (BSA)/PBS for 30 min, the cells were incubated with primary antibodies in 1% BSA/PBS for 1 h. Then, the cells were washed with PBS and incubated with Alexa 488 labeled anti-rabbit secondary antibody for 1 h. Cells were washed and Glass slides were mounted using Superfrost (Matsunami, Japan). Images were obtained using fluorescence microscope (BZ-9000, KEYENCE, Japan).

### 2.6 Mice

*p32*^*flox/flox*^ mice have been described elsewhere (Yagi et al., 2012). *LysM-Cre* [B6.129P2-*Lyz2*^*tm1(cre)Ifo*^/J] mice were purchased from The Jackson Laboratory, USA. Sex-matched *p32*^*flox/flox*^/*LysM-Cre* + and littermate control *p32*^*flox/flox*^/*LysM-Cre*- (wild type, WT) were used for all experiments in this study. Mice were kept under specific pathogen-free conditions in the animal facility of Kyushu University. Animal protocols were approved by the committee of Ethics on Animal Experiment, Faculty of Medical Sciences, Kyushu University.

### 2.7 LPS-induced Endotoxin Shock

WT and *p32*^*flox/flox*^/*LysM-Cre* + mice were age- and sex-matched. Mice were anesthetized and intraperitoneally injected with LPS (25 mg/kg body weight) at 8–12 weeks of age. Survival of the mice was monitored for up to 7 days after injection. For serum cytokine assay, sera were collected at various time points after the LPS injection. Liver were also obtained at 6 and 24 h for histological analysis. ELISA of mouse IL-6 was performed using Mouse IL-6 ELISA Ready-SET-GO!® (eBioscience, USA), according to the manufacturer's instructions.

### 2.8 Flow Cytometry

PE-conjugated anti-mouse CD284 (TLR4) (SA15-21) and PE-conjugated anti-mouse CD14 (Sa14-2) were purchased from Biolegend, USA. FITC-conjugated anti-mouse F4/80 (BM8) and PE-conjugated anti-mouse CD11b (M1/70) were obtained from Bay bioscience, Japan. Before staining with the antibodies, cells were incubated for 10 min on ice with anti-Fcγ III/II receptor (2.4G2; BD Biosciences, USA) antibody to block Fc receptors. Flow cytometry analysis was performed by FACS Verse using FACSuite Software (BD Biosciences, USA).

### 2.9 Statistical Analysis

Data are expressed as means ± SD or means ± SEM of the indicated number of replicates. Statistical analyses of the data were performed using two-tailed Student's *t*-test or log-rank test, and significant differences were based on P < 0.05.

## 3 Results

### 3.1 p32 Ablation Markedly Enhances IL-6 Production in MEFs

Because our previous paper demonstrated that p32-deficient mice died during embryonic development owing to loss of mitochondrial protein translation, we prepared MEFs from wild type (WT) and p32-deficient (p32^−/−^) embryonic mice (Yagi et al., 2012). Mitochondrial p32 is reported as a negative regulator of antiviral immunity (Xu et al., 2009b) (Wang et al., 2014b) (Liu et al., 2015). Consistent with a previous paper (Xu et al., 2009b), *Il-6* and *Ifnb1* mRNA levels were significantly higher in p32 deficient MEFs after TLR3 ligand polyinosinic polycytidylic acid (poly IC) stimulation (Fig. S1).

**Fig. 1:**
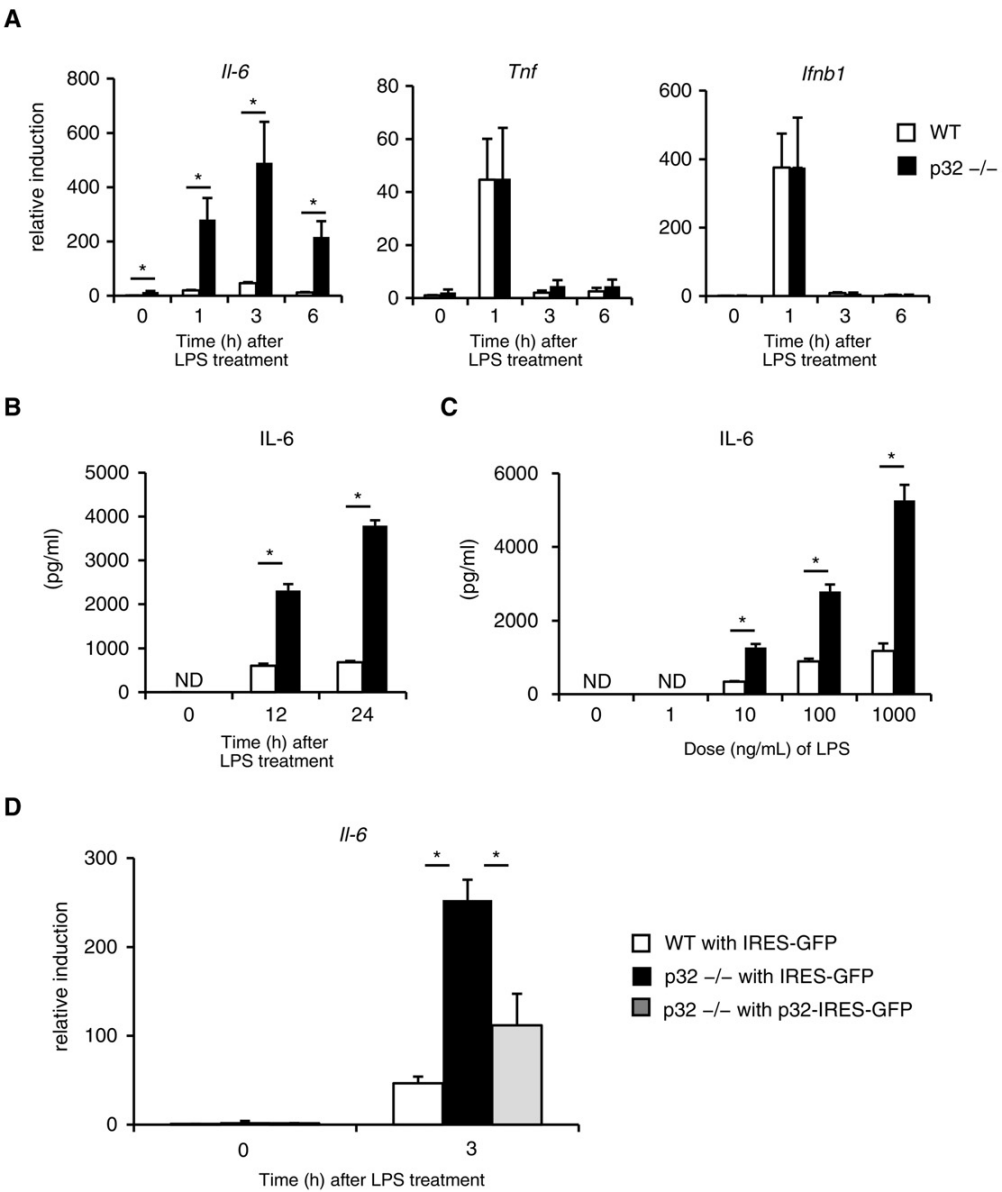
The role of p32 in response to LPS. (A) Real-time PCR analysis of *Il-6*, *Tnf* and *Ifnb1* expression in MEFs stimulated with 100 ng/mL LPS for the indicated time periods. Data are expressed as the mean ± SEM after normalization to expression of the gene encoding *18S* ribosomal RNA (*18S* rRNA) and are representative of two independent experiments. (B.C) The levels of IL-6 in cell culture supernatants were compared between WT and p32^−/−^ MEFs (2 × 10^5^/well) after 100 ng/mL LPS stimulation for the indicated time periods (B), and 24 h after stimulation with the indicated dose of LPS (ng/mL) (C). Data are expressed as the mean ± SD of triplicate wells and are representative of two independent experiments. ND indicates below the detectable limit. (D) Real-time PCR analysis of *Il-6* expression in MEFs stimulated with LPS. MEFs were retrovirally transduced to express either IRES–GFP (empty) or WT p32–IRES–GFP. After fluorescence-activated cell sorting, GFP-positive cells were stimulated with 100 ng/mL LPS for 3 h. Data are expressed as the mean ± SEM after normalization to expression of the gene encoding *18S* rRNA and are representative of two independent experiments. (A–D) *, P < 0.05.

Because the role of p32 in antibacterial response has not been examined so far, we first compared the kinetics of cytokine gene expression induced by LPS between WT and p32^−/−^ MEFs. The expression of *Tnf* and *Ifnb1* were comparably upregulated in response to LPS in both types of MEFs (Fig. 1A). However, MEFs lacking p32 showed drastically (around ten-fold) increased gene expression of *Il-6* compared with WT after LPS stimulation (Fig. 1A). We also found the expression of *Il-6* mRNA significantly increased in p32^−/−^ MEFs even under unstimulated conditions (i.e., at time zero in Fig. 1A).

IL-6 protein levels in the supernatant were below a detection limit without LPS (Fig. 1B, C). In contrast, LPS stimulation significantly upregulated IL-6 production with a time- and dose-dependent effect in p32^−/−^ MEFs (Fig. 1B, C). To establish whether the loss of p32 is involved in LPS-induced IL-6 production, we retrovirally transduced p32^−/−^ MEFs to express p32. Having found that p32 re-expression reduced the transcription of *Il-6* in p32^−/−^ MEFs after LPS stimulation (Fig. 1D), we confirmed that the loss of p32 enhanced LPS-induced IL-6 production in MEFs.

### 3.2 p32 Deficiency Does Not Affect LPS-induced Activation of the NF-κB Pathway.

**Fig. 2:**
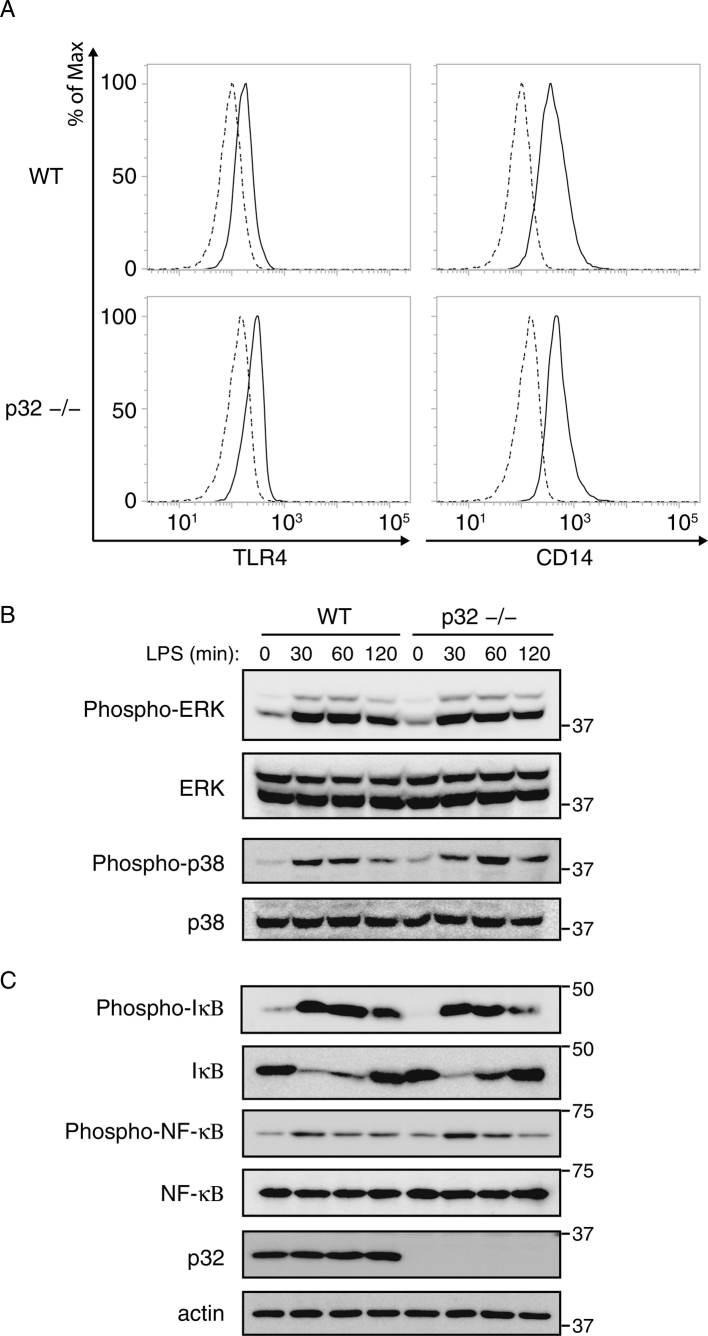
NF-κB signaling are unaffected in the absence of p32. (A) FACS analysis of TLR4 (CD284) and CD14 in MEFs. IgG isotype control is shown as a dotted line. (B.C) MEFs were stimulated with 100 ng/mL LPS for the indicated time periods and analyzed for phosphorylation of ERK, p38 (B), NF-κB, or IκB (C). Data are representative of at least two independent experiments.

To explore the mechanism by which p32 controls IL-6 induction during LPS stimulation, we compared cell surface receptors between WT and p32^−/−^ MEFs. Because both types of MEFs equally expressed TLR4 and CD14 (Fig. 2A), we thought that this overproduction was not a consequence of receptor expression. TLR4-induced activation of signaling cascades of the transcription factor NF-κB and mitogen-activated protein kinases (MAPKs) contribute to inflammatory gene activation (Akira et al., 2006). However, p32 deficiency did not affect TLR4-induced activation of the MAPKs, namely p38 and extracellular signal-regulated kinase (ERK) (Fig. 2B). Similarly, loss of p32 did not affect LPS-induced degradation of IκB or phosphorylation of IκB and NF-κB (Fig. 2C). These results suggest that LPS-induced activation of IκB and NF-κB is unaffected by the absence of p32 in MEFs.

Because p32 did not affect the canonical NF-κB pathway after LPS stimulation (Fig. 2), we further explored the role of p32 in LPS-induced IL-6 overproduction. Previous reports showed that *Il-6* mRNA is destabilized by regnase-1 (also known as Zc3h12a and MCPIP1) in response to LPS in macrophages (Iwasaki et al., 2011, Matsushita et al., 2009). Then, we examined the stability of *Il-6* mRNA in MEFs. Although *Il-6* mRNA was slightly stable in p32^−/−^ MEFs treated with LPS (Fig. S2), we concluded that p32 is not an important factor associated with the stability of *Il-6* mRNA.

### 3.3 Loss of p32 Increases LPS-induced ATF4 Activation and Nuclear Translocation

**Fig. 3:**
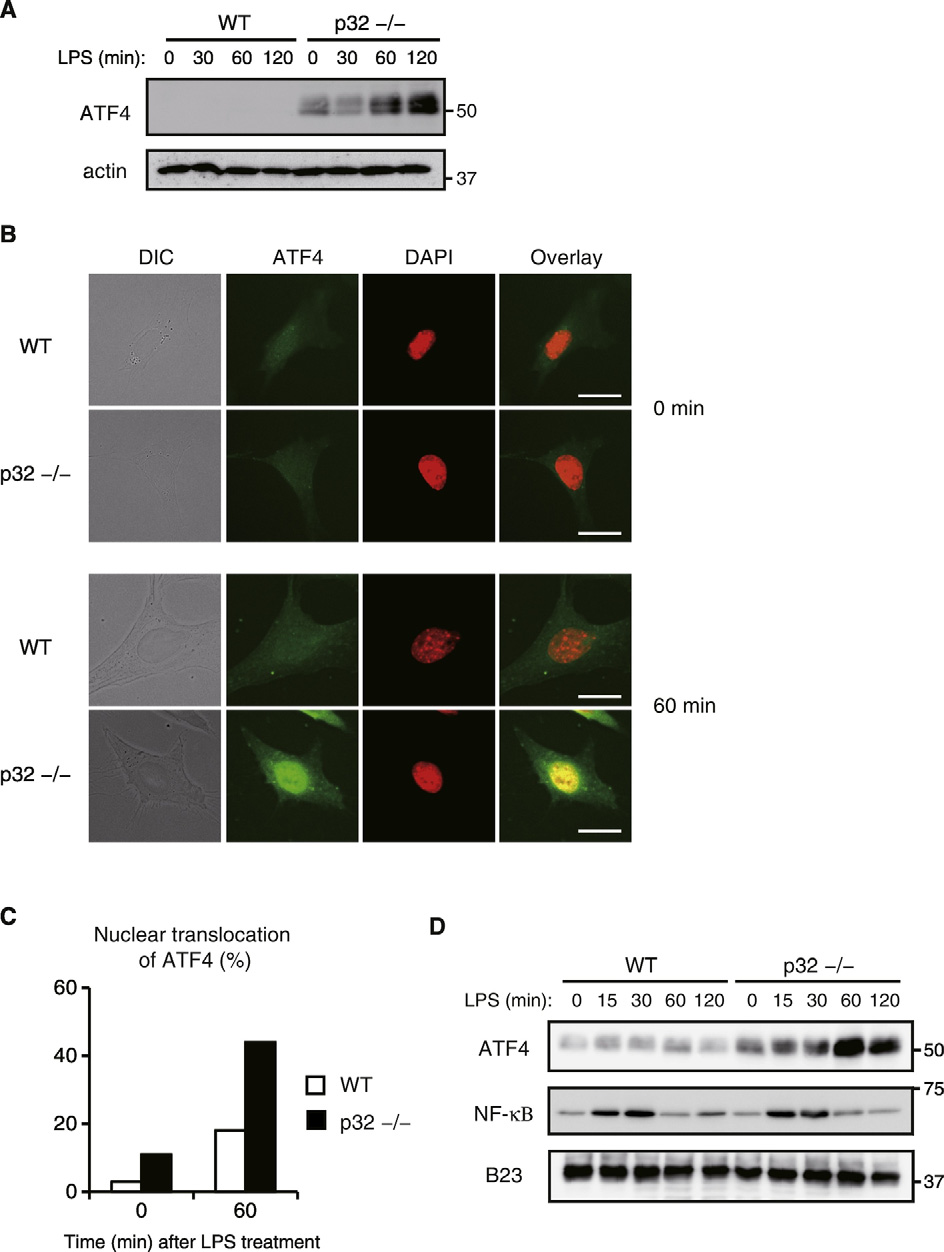
Subcellular localization of ATF4 in MEFs. (A) MEFs were stimulated with 100 ng/mL LPS for the indicated time periods. Total cell lysates were analyzed by Western blotting for ATF4 expression. (B·C) Subcellular localization of ATF4 was compared between WT and p32^−/−^ MEFs after stimulation with LPS (100 ng/mL). DAPI was used to stain nuclei. Representative images of three independent experiments are shown. Bar, 20 μm (C) The percentage of cells in which ATF4 merged with DAPI is shown. Data are representative of at least two independent experiments. (D) MEFs were stimulated with LPS for the indicated times. Nuclear proteins were prepared and subject to immunoblot analysis using anti-ATF4 and anti-NF-κB. Nucleophosmin (B23) proteins were used as nucleolus markers.

Activating transcription factor 4 (ATF4) is induced by endoplasmic reticulum (ER) stress, metabolic stress, and mitochondrial stress (Harding et al., 2000) (Iwasaki et al., 2014) (Bouman et al., 2011) (Kim et al., 2013). In addition, a recent study demonstrated that ATF4 directly activates *Il-6* transcription and acts in synergy with the TLR4 pathway (Iwasaki et al., 2014). For identifying how p32 controls LPS-induced IL-6 production, we next investigated whether p32 is involved in an ATF4 dependent pathway. In WT MEFs, ATF4 was not significantly upregulated in response to LPS (Fig. 3A). In contrast, we found that LPS stimulation drastically increased the expression of ATF4 in p32^−/−^ MEFs (Fig. 3A). ATF4 is translocated from the cytoplasm to the nucleus and directly binds to the *Il-6* promoter region at the cAMP response element in response to various stimulations (Zhang et al., 2013) (Iwasaki et al., 2014). Thus, we next examined the nuclear translocation of NF-κB and ATF4 by staining MEFs with anti-NF-κB and anti-ATF4 antibodies together with DAPI-staining of nuclei. NF-κB was equally expressed in the nucleus of both types of MEFs in response to LPS (Fig. S3). However, stimulation with LPS induced nuclear accumulation of ATF4 in p32^−/−^ but not WT MEFs (Fig. 3B, C). Similarly, the protein levels of ATF4 in the nucleus much more increased in p32^−/−^ MEFs than WT MEFs. Based on these observations, we thought that LPS activates only the NF-κB signaling pathway in WT MEFs. In contrast, in p32^−/−^ MEFs, ATF4 is also activated and translocated to the nucleus by LPS stimulation in parallel with the nuclear translocation of NF-κB.

### 3.4 ATF4 is Required for IL-6 Overproduction in p32^−/−^ MEFs

**Fig. 4:**
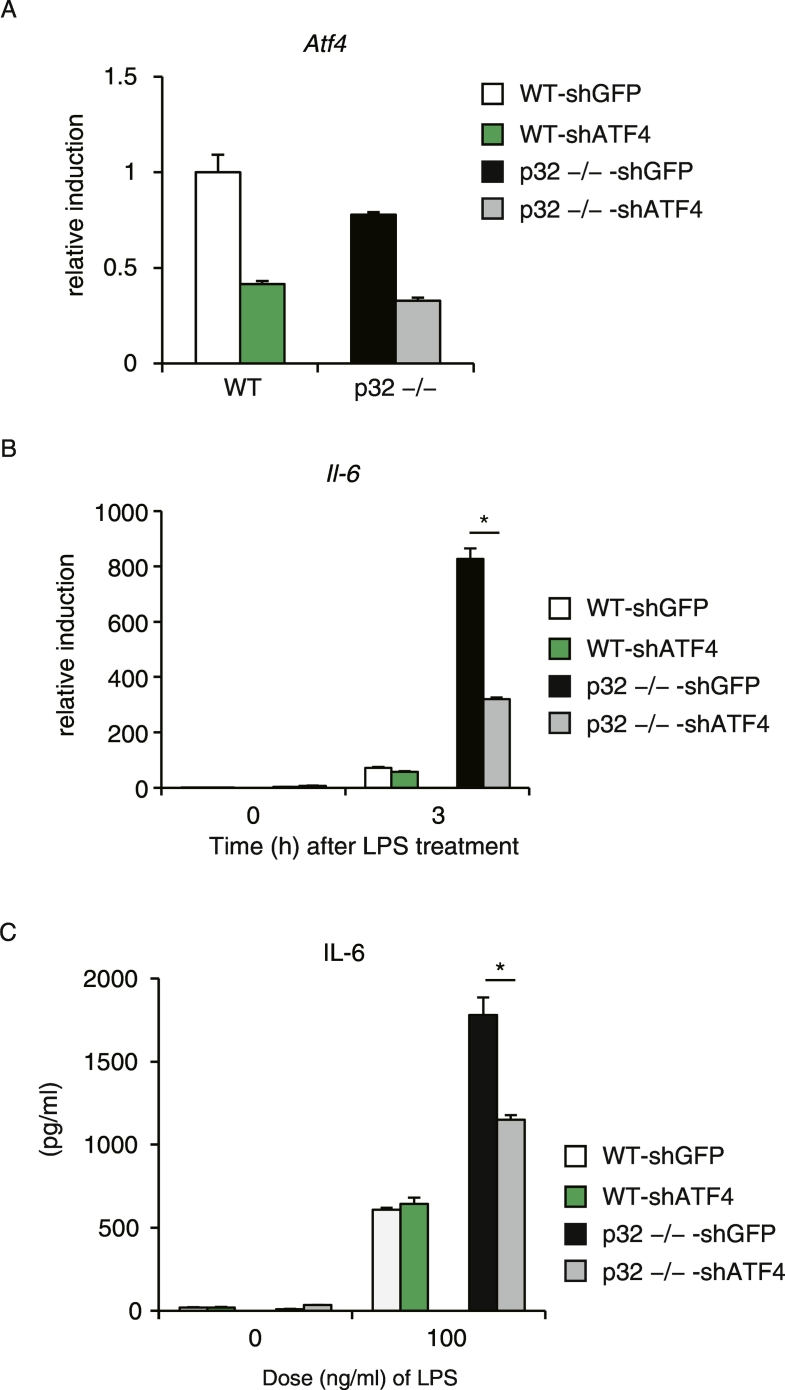
ATF4 controls LPS-induced IL-6 overproduction in p32^−/−^ MEFs. (A) Expression of Atf4 mRNA in MEFs transduced with retroviral vector expressing control shGFP or *Atf4*-specific shRNA. (B) Real-time PCR analysis of *Il-6* expression in MEFs stimulated with 100 ng/mL LPS for 3 h. Data are expressed as the mean ± SD of triplicate reactions after normalization to expression of the gene encoding *18S* rRNA and are representative of two independent experiments. (C) The levels of IL-6 in cell culture supernatants are compared between MEFs stimulated with 100 ng/mL LPS for 24 h. Data are expressed as the mean ± SD of triplicate wells and are representative of two independent experiments. *, P < 0.05 (B.C).

We have demonstrated that loss of p32 increases LPS-induced IL-6 production (Fig. 1) and ATF4 activation (Fig. 3). To examine the direct impact of ATF4 pathway activation on IL-6 production in MEFs in response to LPS, we retrovirally knocked down *Atf4*, which was confirmed by quantitative PCR (Fig. 4A). *Atf4* knockdown reduced LPS-induced *Il-6* expression in p32^−/−^ MEFs (Fig. 4B.C). On the other hand, there was no significant difference in LPS-induced *Il-6* expression in WT MEFs (Fig. 4B, C). These results further indicate that ATF4 is required for LPS-induced IL-6 production in p32^−/−^ MEFs but not in WT.

Several reports demonstrate that ATF4 is induced by saturated fatty acid (e.g. palmitate) (Iwasaki et al., 2014) (Wang et al., 2014a). As LPS-induced IL-6 overproduction required the ATF4 signaling pathway in p32^−/−^ MEFs (Fig. 4B, C), we examined the amounts of fatty acids in WT and p32^−/−^ MEFs. Fatty acid analysis showed that the amount of palmitate did not significantly increase in p32^−/−^ MEFs, compared to WT MEFs (Fig. S4A). In addition, we did not find any significant difference in the amount of other fatty acids and the fluorescence intensity of boron-dipyrromethene (BODIPY) fatty acid analog between WT and p32^−/−^ MEFs (Fig. S4B). As Etomoxir, an inhibitor of FAO, did not enhance LPS-induced *Il-6* expression (Fig. S4C), we concluded LPS-induced IL-6 overproduction in p32^−/−^ MEFs was not dependent on the amount of fatty acids and FAO.

### 3.5 Inhibition of Mitochondrial Translation Increases LPS-induced ATF4 Activation and IL-6 Production

Mitochondrial p32 binds to mitochondrial RNAs in mitochondrial ribosomes and regulates mitochondrial protein synthesis (Yagi et al., 2012). Given our finding that the loss of p32 induced IL-6 production via ATF4 activation during LPS stimulation, we hypothesized that mitochondrial translation might influence IL-6 secretion.

**Fig. 5:**
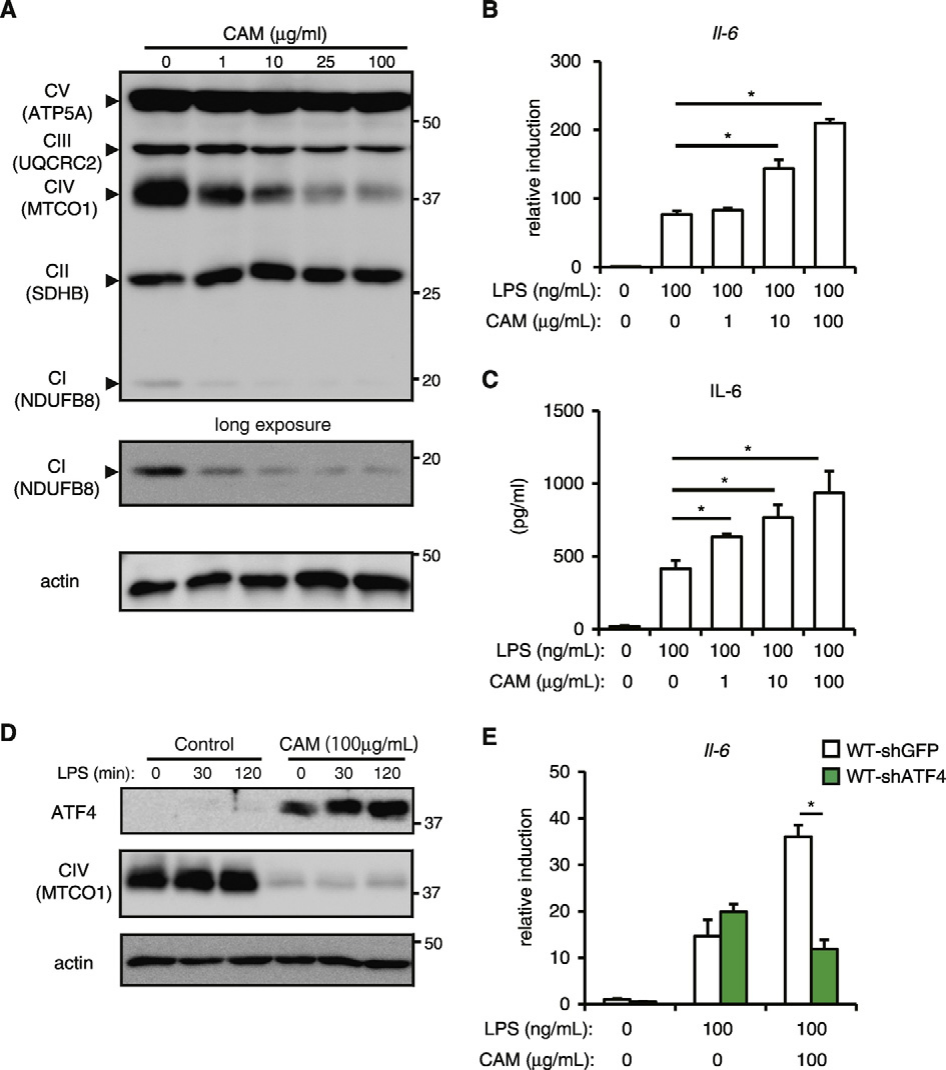
Inhibition of mitochondrial translation upregulates LPS-inducued IL-6 induction in MEFs. (A)Western blot analysis of mitochondrial respiratory complexes in MEFs treated with the indicated chloramphenicol concentration. (B) Real-time PCR analysis of Il6 expression in MEFs treated with the indicated chloramphenicol concentration and stimulated with 100 ng/mL LPS for 3 h. Data are expressed as the mean ± SD of triplicate reactions after normalization to expression of the gene encoding *18S* rRNA and are representative of two independent experiments. (C) IL-6 production were compared among WT MEFs treated with the indicated chloramphenicol concentration. Cells were stimulated with 100 ng/mL LPS for 24 h in the presence or absence of inhibitor. Data are expressed as the mean ± SD of triplicate wells and are representative of two independent experiments. (D) MEFs in the presence or absence of chloramphenicol were stimulated with 100 ng/mL LPS for the indicated times and analyzed for the level of ATF4. (E) Real-time PCR analysis of *Il6* expression in MEFs transduced with retroviral vector expressing control shGFP or *Atf4*-specific shRNA. Cells were stimulated with 100 ng/mL LPS for 3 h. Data are expressed as the mean ± SD of triplicate reactions after normalization to expression of the gene encoding *18S* rRNA and are representative of two independent experiments. *, P < 0.05 (B.C.E).

Chloramphenicol (CAM) is an inhibitor of mitochondrial translation (Richter et al., 2013). CAM (over 25 μg/mL) decreased the mitochondrial encoded core subunit of cytochrome oxidase mt-COX1 to almost undetectable levels, but there was no difference in the expression level of nuclear-encoded complex II 70 kDa subunit or ATP synthase 5A in response to CAM. Moreover, ubiquinone oxidoreductase subunit B8, a subunit of complex I, was also impaired in MEFs with CAM (Fig. 5A). These observations were consistent with the protein levels of p32^−/−^ MEFs in response to CAM (Yagi et al., 2012).

We then investigated whether mitochondrial translation is involved in the increased IL-6 production in response to LPS. MEFs activated with LPS in the presence of CAM showed increased levels of *Il-6* mRNA two- to three-fold (Figs. 5B). Additionally, we found CAM caused dose-dependent production of IL-6 in MEFs with LPS (Fig. 5C). A similar result was obtained in response to actinonine (Fig. S5A). We next examined whether mitochondrial translation inhibition is associated with ATF4 activation. ATF4 was upregulated in response to LPS with CAM in MEFs (Fig. 5D), similar to p32^−/−^ MEFs. Moreover, ATF4 knockdown reduced LPS-induced IL-6 production in the presence of CAM to the control level (Fig. 5E). These results indicate that inhibition of mitochondrial translation also controls TLR4-mediated IL-6 induction in MEFs through ATF4 activation, similar to the loss of p32.

Having found that inhibition of mitochondrial translation enhanced LPS-induced IL-6 production, we hypothesized that other mitochondrial inhibitors might also increase the production of IL-6. As several papers suggest that mitochondrial respiratory chain is a central determinant of clinical outcome among septic patients (Brealey et al., 2002) (Brealey et al., 2004), we next investigated several mitochondrial respiratory chain inhibitors in MEFs. Consistent with the experiments using CAM, several inhibitors including rotenone, a mitochondrial complex I inhibitor; antimycin A, a mitochondrial complex III inhibitor; and oligomycin, a mitochondrial complex V inhibitor, enhanced the levels of *Il-6* mRNA in MEFs with LPS (Fig. S5B). In contrast, treatment with carbonyl cyanide *m*-chlorophenylhydrazone (CCCP), a protonophore that dissipates mitochondrial membrane potential by increasing membrane permeability to protons, did not affect LPS-induced *Il-6* mRNA in WT MEFs (Fig. S5B). Mitochondrial membrane potential had only a slight effect on LPS-induced IL-6 overproduction in contrast to antiviral response (Koshiba et al., 2011). These observations suggest that mitochondrial respiratory chain inhibitors also enhance TLR4-mediated IL-6 induction in MEFs, similar to the loss of p32 and inhibition of mitochondrial translation.

### 3.6 p32 Protects Mice from Endotoxin Shock

**Fig. S6:**
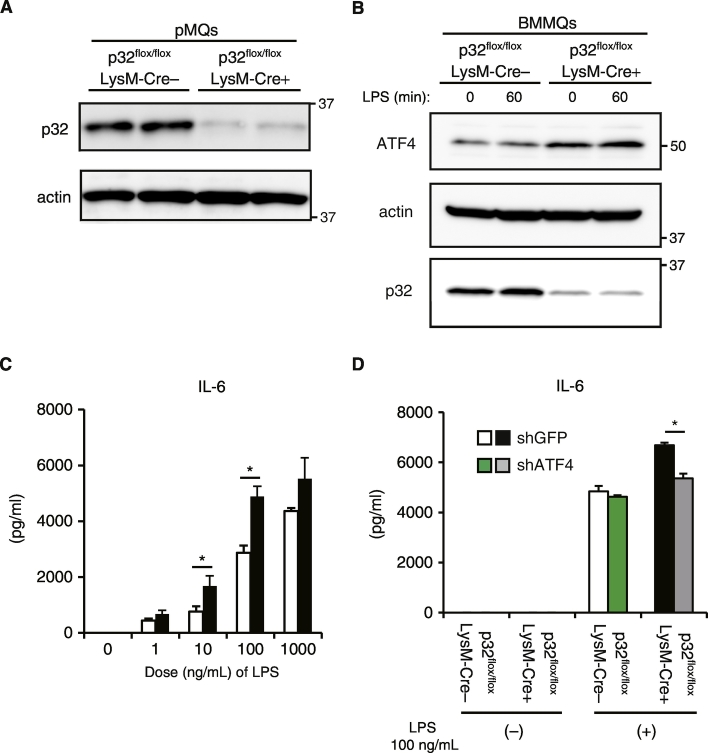
Confirmation of the loss of p32 protein in macrophages from *p32flox/flox*/*LysM-Cre +* mice. (A) Western blot analysis of p32 in pMQs from *p32flox/flox*/*LysM-Cre +* mice and *p32flox/flox*/*LysM-Cre-* mice. (B) Western blot analysis of p32 and ATF4 in BMMQs from *p32flox/flox*/*LysM-Cre +* mice and *p32flox/flox*/*LysM-Cre-* mice. (C) The levels of IL-6 in cell culture supernatants were compared between BMMQs (4 × 104/well) from *p32flox/flox*/*LysM-Cre +* mice and *p32flox/flox*/*LysM-Cre-* mice 24 hours after stimulation with the indicated dose of LPS (ng/mL). (D) The levels of IL-6 in cell culture supernatants are compared between BMMQs transduced with retroviral vector expressing control shGFP or Atf4-specific shRNA with 100 ng/mL LPS for 24 hours. Data are expressed as the mean ± SD of triplicate wells and are representative of two independent experiments. *, P < 0.05

Our data indicated that p32 is critically involved in LPS-induced IL-6 expression in MEFs (Fig. 1). To examine the role of p32 in TLR4-mediated cytokine production in vivo, we generated a macrophage/neutrophil-specific p32 conditional knockout mouse by mating loxP-flanked p32 gene mutation *p32*^*flox/flox*^ mice with *lysozyme M-Cre* transgenic mice. Western blot analyses indicated almost complete loss of p32 in peritoneal macrophages (pMQs) and BMMQs derived from *p32*^*flox/flox*^/*LysM-Cre* + mice (Fig. S6A and B). Flow cytometry analysis after intracellular staining with p32 antibody revealed that the expression of p32 was decreased in F4/80 + CD11b + macrophage from *p32*^*flox/flox*^/*LysM-Cre* + mice (data not shown). Moreover, BMMQs lacking p32 showed increased production of IL-6 (around 1.7 times) after LPS stimulation (10–100 ng/mL) (Fig. S6C). Because ATF4 protein levels were increased in BMMQs from *p32*^*flox/flox*^/*LysM-Cre* ± mice, compared to *p32*^*flox/flox*^/*LysM-Cre-* (Fig. S6B), we retrovirally knocked down *Atf4* to examine the impact of ATF4 on IL-6 production in BMMQs in response to LPS. Indeed, *Atf4* knockdown significantly reduced LPS-induced IL-6 production in BMMQs from *p32*^*flox/flox*^/*LysM-Cre* ± mice (Fig. S6D), consistent with MEFs (Fig. 4C).

**Fig. 6:**
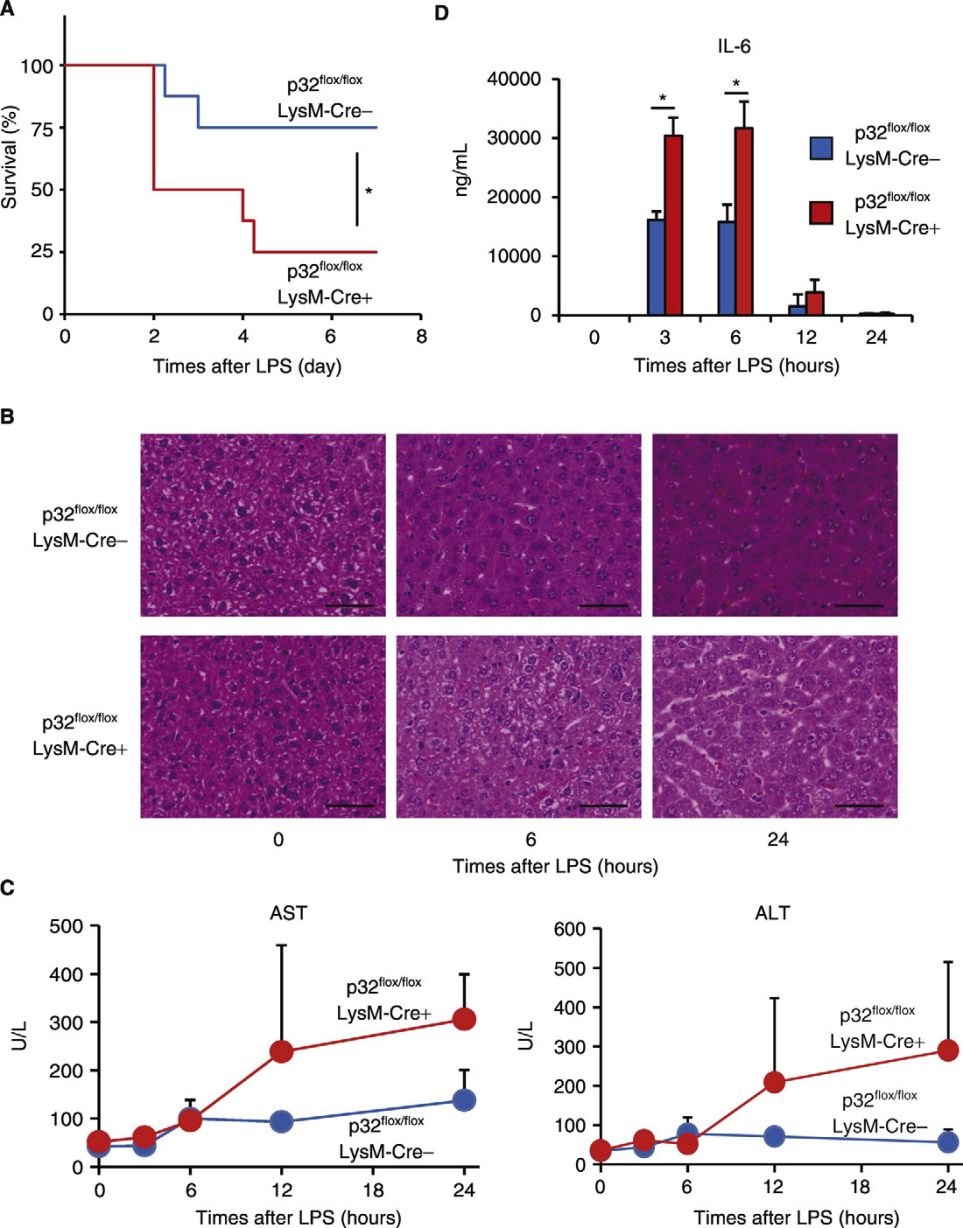
p32 affects LPS-induced IL-6 production in vivo. (A) Kaplan-Meier plot of Age-matched *p32*^*flox/flox*^/LysM-Cre + and wild-type control (*p32*^*flox/flox*^/LysM-Cre-) mice (*n* = 8) treated with LPS (25 mg/kg). (B) Representative H&E staining of liver sections in *p32*^*flox/flox*^/LysM-Cre + and wild-type control mice. (C) Serum levels of ALT and AST from *p32*^*flox/flox*^/LysM-Cre + and wild-type control mice for the indicated times. (D) Serum IL-6 levels were measured by Enzyme-linked immunosorbent assay (ELISA) for the indicated times after LPS challenge (n ≥ 3 mice/group). (A–D) Data are representative of two independent experiments. (A) *P < 0.05, compared with wild-type control mice treated with LPS (log-rank test (A) or two-tailed Student's *t*-test (C.D)).

To examine whether p32 is associated with sepsis progression, we measured the survival rate after intraperitoneally injecting LPS into littermate wild-type controls (*p32*^*flox/flox*^/*LysM-Cre-*) and *p32*^*flox/flox*^/*LysM-Cre* + mice. After 2 days, half of the *p32*^*flox/flox*^/*LysM-Cre* + mice (50%) had died while all of the wild-type control mice survived, (Fig. 6A). In a lower-dose LPS-shock model, similar results were obtained (data not shown). These observations indicate that *p32*^*flox/flox*^*/LysM-Cre* + mice are highly susceptible to LPS-induced endotoxin shock. The exposure to LPS caused multi-organ system failure such as liver, lung, and kidney injury (Szarka et al., 1997) (Hsu et al., 2006). We then analyzed whether the liver of these mice were injured after LPS injection. Although the majority of the livers in wild-type control mice were normal or contained only scattered foci of mild chronic inflammation, LPS caused liver damages including hepatic necrosis in *p32*^*flox/flox*^/*LysM-Cre* + mice (Fig. 6B). Moreover, the intraperitoneal injection of LPS induced a substantial increase in the serum AST and ALT from *p32*^*flox/flox*^/*LysM-Cre* + mice compared with littermate wild-type controls (Fig. 6C).

**Fig. S7:**
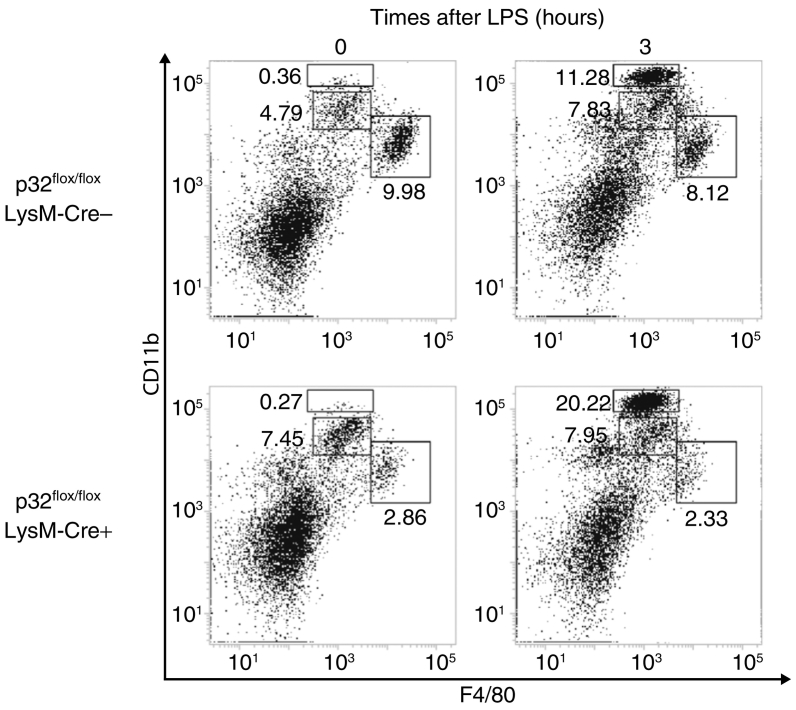
Inflammatory cell infiltration in liver after LPS administration. Liver MNCs were obtained from *p32flox/flox*/*LysM-Cre +* mice and *p32flox/flox*/*LysM-Cre*mice and expression of F4/80 with CD11b were examined. Data are representative of two independent experiments.

Having found that p32 is involved in the LPS-induced sepsis model, we measured serum IL-6 after intraperitoneally injecting LPS. Consistent with the observations in the cultured MEFs (Fig. 1), serum IL-6 was significantly higher in *p32*^*flox/flox*^/*LysM-Cre* + mice than WT after LPS injection (Fig. 6D). We also found that intrahepatic *Il-6* mRNA levels were significantly increased in *p32*^*flox/flox*^/*LysM-Cre* + mice (data not shown). In addition, LPS-induced inflammatory cell infiltration increased in *p32*^*flox/flox*^/*LysM-Cre* + mice (Fig. S7). Collectively, these data demonstrate that p32 also controls LPS-induced IL-6 production in macrophages and protects mice against endotoxin shock in vivo.

## 4 Discussion

The involvement of p32 and mitochondrial function has been suggested in antiviral immunity (West et al., 2011b) (Koshiba, 2013). In contrast, the role of p32 in antibacterial immunity was unknown. In this study, we have demonstrated that the absence of p32 increases LPS-induced IL-6 production both in vivo (Fig. 6) and in vitro (Fig. 1). In addition, our study clearly indicates that the loss of p32 enhances TLR4-mediated IL-6 production via not an NF-κB dependent pathway but an ATF4 dependent pathway in MEFs (Fig. 2, Fig. 3, Fig. 4). Our findings strongly suggest that the ATF4 pathway is activated by mitochondrial stress response caused by the inhibition of mitochondrial translation and then enhances LPS-induced IL-6 production (Fig. 5, Supplemental Fig. 5A). This ATF4 response may be an important pathway linking sepsis-mediated MODS to mitochondrial dysfunction. Mitochondrial antiviral immunity depends on a mitochondrial membrane potential, but not mitochondrial respiratory function (Koshiba et al., 2011). In contrast, we found that the inhibition of mitochondrial respiratory chain, but not depolarization of the membrane potential, enhanced LPS-induced IL-6 production (Supplemental Fig. 5B). Because different TLR ligands such as polyIC and LPS generated different inflammatory responses under same mitochondrial dysfunction, specific cellular systems through mitochondria may be activated by distinct infectious contexts. In this paper, we propose that LPS-induced IL-6 overproduction occurs under specific mitochondrial dysfunctional conditions via ATF4 activation. The fine molecular mechanisms underlying the ATF4 signaling pathways in the p32^−/−^ MEFs remain to be fully elucidated.

The ATF family represents a large group of basic-region leucine zipper (bZIP) transcription factors. ATF4, a member of the ATF family, forms homodimers and heterodimers with other transcription factors. Although ATF4 plays an important role in many inflammatory processes, such as endoplasmic reticulum (ER) stress, metabolic stress, and mitochondrial stress (Harding et al., 2000) (Iwasaki et al., 2014) (Bouman et al., 2011) (Kim et al., 2013), it remains unclear whether ATF4 participates in TLR signaling. We did not observe any significant difference in LPS-induced IL-6 production between negative control and ATF4 knockdown in WT MEFs (Fig. 4B, C), suggesting that LPS-dependent activation of ATF4 does not occur under healthy mitochondrial conditions. Although another group demonstrated that ATF4 is activated and translocates to the nucleus following LPS stimulation via the TLR4-dependent pathway in THP-1 cells (Zhang et al., 2013), we consider that the LPS-dependent activation of ATF4 could have been further augmented under specific unhealthy mitochondrial conditions.

Many researchers have conducted experimental studies on mitochondrial dysfunction in sepsis. When Zheng et al. searched PubMed databases and Web of Science databases from 1950 to January 2014, 121 studies were identified involving the use of mitochondrial therapy in sepsis (Zheng et al., 2015). According to the effects of drugs on different aspects of mitochondria, the authors divided them into four categories: 1. mitochondrial matrix and respiratory chain, 2. mitochondrial antioxidant, 3. mitochondrial membrane stability, and 4. hormone therapy for septic mitochondria. Our observations demonstrate that mitochondrial respiratory chain inhibitors including rotenone, antimycin A and oligomycin enhanced the levels of *Il-6* mRNA in MEFs with LPS (Fig. S5B). These results may relate mitochondrial dysfunction to outcome of septic shock. Indeed, in severe septic patients, mitochondrial complex I activity of skeletal muscle have a significant inverse correlation with therapeutic requirement (Brealey et al., 2002). Brealey et al. also show that muscle adenosine triphosphate (ATP) concentrations are significantly lower in septic non-survivors than in septic survivors (Brealey et al., 2002). Mitochondrial respiratory chain plays a critical part in the biosynthesis of ATP. Although the relationship between mitochondrial respiratory chain and IL-6 is not clear in septic patient, based on the current work, we believe that improvement of mitochondrial respiratory chain is useful for severe septic patient with high concentration of IL-6.

Several studies demonstrate that LPS increases mitochondrial reactive oxygen species (ROS) in macrophages (Bulua et al., 2011) (West et al., 2011a). In addition, pharmacological blockade of mitochondrial ROS efficiently reduces inflammatory cytokine production after LPS stimulation in cells (Bulua et al., 2011). Therefore, we compared mitochondrial ROS production between WT and p32^−/−^ MEFs. As we could not find any significant difference in both types of MEFs (data not shown), we thought mitochondrial ROS production did not affect LPS-induced IL-6 overproduction in p32^−/−^ MEFs.

Here, we propose a fifth category, that is the inhibition of mitochondrial translation, which relates to mitochondrial dysfunction in sepsis. Linezolid, another type of mitochondrial translation inhibitor, significantly increases the expression of *Il-6* in co-stimulation with LPS (Bode et al., 2015). These results, including our data (Fig. 5) provide direct evidence that mitochondrial translation inhibitors modulate cytokine production, including IL-6.

In the current study, we identified that p32 prevents overproduction of LPS-induced IL-6 (Fig. 1). The *Il6* mRNA expression was upregulated by LPS in the presence of mitochondrial translation inhibitors in MEFs but to a lesser extent than the p32 absence (compare Figs. 1A and 5B). The *Atf4* knockdown in p32^−/−^ MEFs did not decrease IL-6 mRNA to a WT level (Fig. 4B) while the knockdown in the CAM-treated MEFs did to the non-treated cell level (Fig. 5E). Because p32 is proposed as a general RNA and protein chaperon in mitochondria (Yagi et al., 2012), we speculate the possibility that p32 suppresses TLR4-dependent IL-6 production through not only simply maintaining mitochondrial translation but also still unknown factors. Several studies have identified p32 interacting proteins using proteomics analysis so far (Yagi et al., 2012) (Chen et al., 2016). Analysis of p32 interacting proteins may lead to candidate factors for the pathology of sepsis.

We analyzed LPS-induced IL-6 production in vitro mainly in MEFs. MEFs express high levels of mRNA for TLR2 and TLR4. In addition, MEFs are highly responsive to TLR-ligand activation and can produce high levels of IL-6 in response to TLR ligands. MEFs from mice with targeted deletions of TLR4 and MyD88 demonstrated profound defects in their IL-6 response to LPS, consistent with studies of macrophages (Kurt-Jones et al., 2004). We also show that BMMQs are ATF4 dependent similarly to MEFs (Figs. S6B–D). Although MEFs are thus a good model for IL-6 responses, the study on p32 is necessary for other immune cells including dendritic cells and lymphocytes.

There are several reports showing that critically ill patients with sepsis experience a significant increase in the levels of extracellular histones (Zeerleder et al., 2003) (Xu et al., 2009a). Because p32 directly binds to extracellular histones and neutralizes the harmful features of histones, treatment with recombinant p32 significantly improves survival in mice receiving a lethal dose of histones (Westman et al., 2014). Moreover, we found that macrophage/neutrophil-specific p32 conditional knockout mice showed exacerbated inflammation and reduced survival in response to LPS. Based on these findings, p32 is an important regulator of inflammatory signaling and is a potential drug candidate for treatment of sepsis in mammals. In addition, p32 could be a therapeutic target for controlling LPS-related inflammatory diseases in humans.

The following are the supplementary data related to this article.

Fig. S1 p32 ablation enhances poly IC–induced *Il6* mRNA transcription in MEFs.
Real-time PCR analysis of *Il-6*, and *Ifnb1* expression in MEFs stimulated with 100 ng/mL poly IC for the indicated times. Data are expressed as the mean ± SD of triplicate reactions after normalization to expression of the gene encoding *18S rRNA* and are representative of two independent experiments. *, P < 0.05. Image 1

Fig. S2 Differences in the control of IL-6 mRNA stability by LPS.
Quantitative PCR analysis of *Il-6* mRNA among total RNA from WT and p32 −/− MEFs stimulated for 3 h with 100 ng/mL LPS, followed by treatment for 0–4 hours (horizontal axis) with actinomycin D (ActD). Data are expressed as the mean of duplicate reactions after normalization to expression of the gene encoding *18S rRNA* and are representative of three independent experiments. Image 2

Fig. S3 Subcellular localization of NF-κB.
(Upper panel) Subcellular localization of NF-κB (Green) was compared between WT and p32 −/− MEFs after stimulation with LPS. DAPI (Blue) was used to stain nuclei. Representative images of three independent experiments are shown. DIC, Bar, 10 μm. (Lower panel) Percentage of cells that exhibited p65 nuclear translocation. Data represent the mean of two independent experiments. Image 3

Fig. S4 Effect of Etomoxir on LPS − induced *Il6* mRNA levels.
(A) The levels of intracellular fatty acid were measured by mass spectrometry. Data are expressed as the mean ± SD of triplicate measurements. All data are normalized to the average values from WT MEFs. (B) Flow cytometry quantification of lipid accumulation. Unstained cells were used as a negative control (dotted line). Solid line represent MEFs stained with DODIPY, two independent experiments with similar results. (C) Before assay, WT MEFs were pretreated for 24hours with the indicated dose of Etomoxir. Realtime PCR analysis of *Il-6* expression in MEFs stimulated with 100 ng/mL LPS for 3 hours. Data are expressed as the mean of duplicate reactions after normalization to expression of the gene encoding *18S rRNA* and are representative of two independent experiments. Image 4

Fig. S5 Effect of actinonin on LPS − induced *Il6* mRNA levels.
(A) Before assay, WT MEFs were pretreated for 3 h with the indicated dose of actinonin. (B) Before assay, WT MEFs were pretreated for 3 h with the indicated dose of several mitochondrial inhibitors. (A,B) Real-time PCR analysis of *Il6* expression in MEFs stimulated with 100 ng/mL LPS for 3 hours. Data are expressed as the mean of duplicate reactions after normalization to expression of the gene encoding *18S rRNA* and are representative of two independent experiments. Image 5

## Funding Sources

This study was supported by a Grant-in-Aid for Scientific Research from the Japan Society for the Promotion of Science (JSPS; grant numbers #16K19196, #26860512 #25893162 to K.G., #15H04764, #24590387 to T.U., and #17H01550, #25253041 to D.K.). This work was partly supported by research funds from LSI Medience Corporation.

## Competing Financial Interests

The authors declare no competing financial interests.

## Author Contributions

T.U. and D.K. designed the study. K.S., K.G., S.M., D.S., M.Y. and K.I. carried out experiments. Figures were prepared by K.S. and K.G.. K.G. and D.K. wrote the manuscript. D.K. made review and supervised all experiments.
