# Supplementary figures and images for: p32 is Required for Appropriate Interleukin-6 Production Upon LPS Stimulation and Protects Mice from Endotoxin Shock
Source: eBioMedicine. 2017 May 11;20:161–72. doi: 10.1016/j.ebiom.2017.05.018 (PMC5478242; doi:10.1016/j.ebiom.2017.05.018)

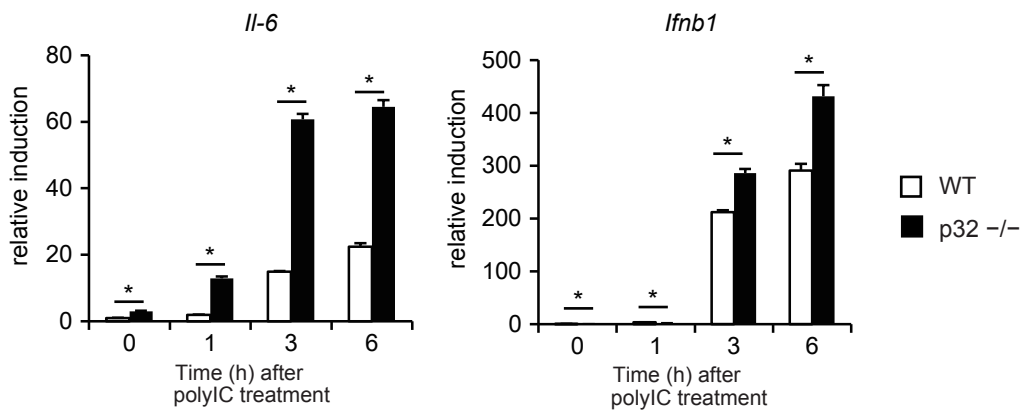

Supplement: Fig. S1 — p32 ablation enhances poly IC–induced Il6 mRNA transcription in MEFs. Real-time PCR analysis of Il-6, and Ifnb1 expression in MEFs stimulated with 100 ng/mL poly IC for the indicated times. Data are expressed as the mean ± SD of triplicate reactions after normalization to expression of the gene encoding 18S rRNA and are representative of two independent experiments. *, P < 0.05. [file mmc1.pdf]

*Il-6*

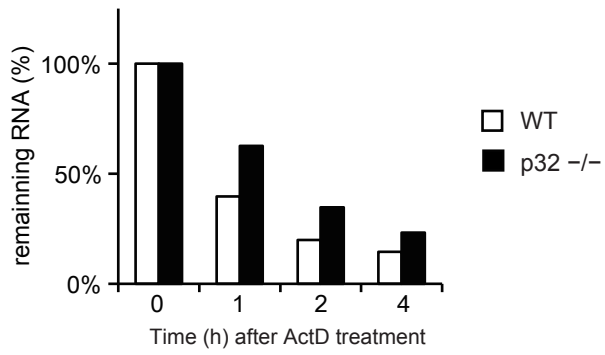

Supplement: Fig. S2 — Differences in the control of IL-6 mRNA stability by LPS. Quantitative PCR analysis of Il-6 mRNA among total RNA from WT and p32 −/− MEFs stimulated for 3 h with 100 ng/mL LPS, followed by treatment for 0–4 hours (horizontal axis) with actinomycin D (ActD). Data are expressed as the mean of duplicate reactions after normalization to expression of the gene encoding 18S rRNA and are representative of three independent experiments. [file mmc2.pdf]

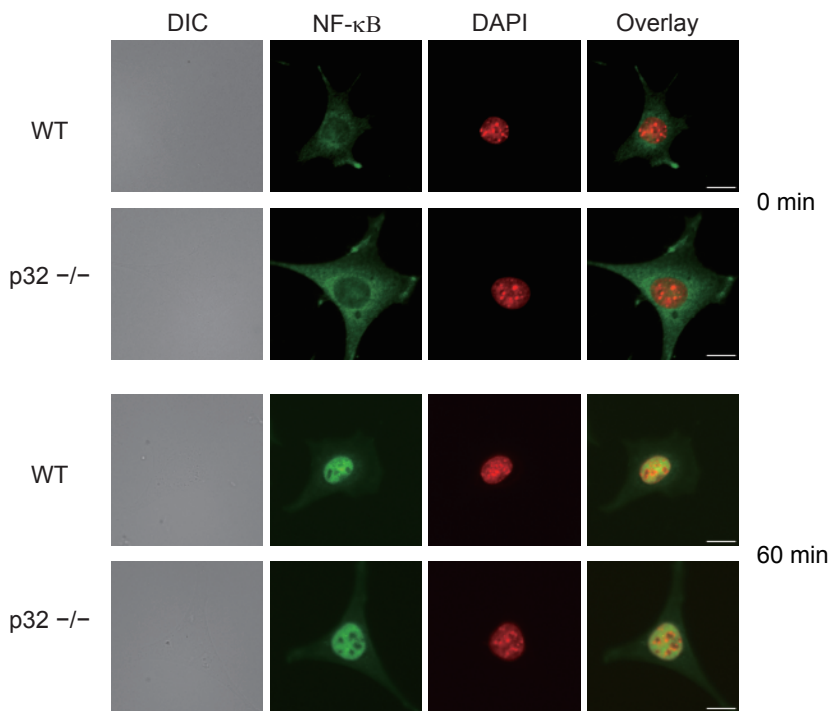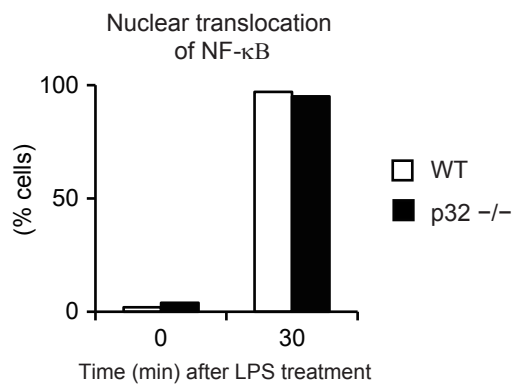

Supplement: Fig. S3 — Subcellular localization of NF-κB. (Upper panel) Subcellular localization of NF-κB (Green) was compared between WT and p32 −/− MEFs after stimulation with LPS. DAPI (Blue) was used to stain nuclei. Representative images of three independent experiments are shown. DIC, Bar, 10 μm. (Lower panel) Percentage of cells that exhibited p65 nuclear translocation. Data represent the mean of two independent experiments. [file mmc3.pdf]

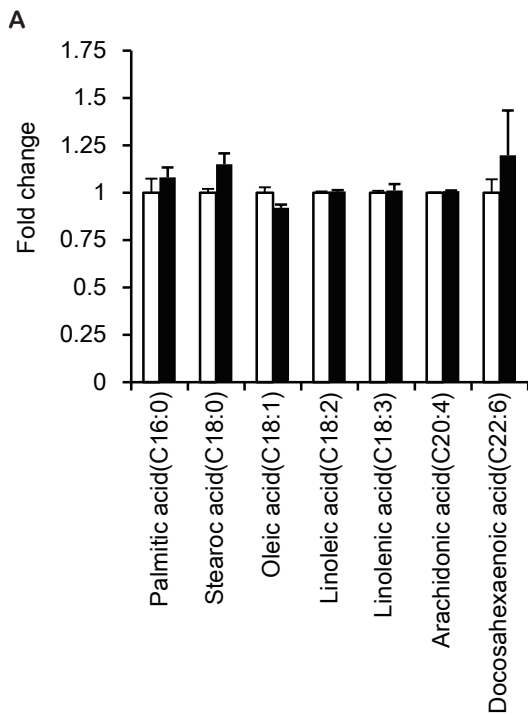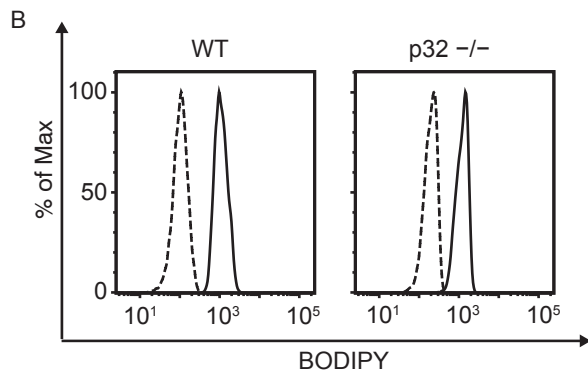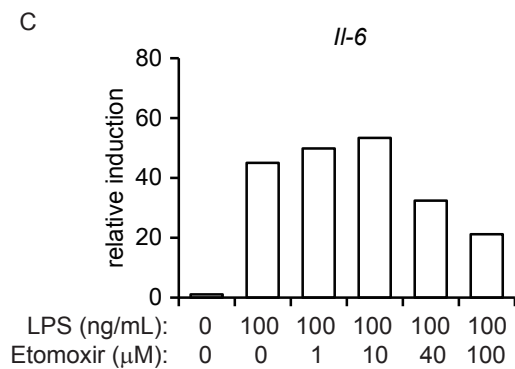

Supplement: Fig. S4 — Effect of Etomoxir on LPS − induced Il6 mRNA levels. (A) The levels of intracellular fatty acid were measured by mass spectrometry. Data are expressed as the mean ± SD of triplicate measurements. All data are normalized to the average values from WT MEFs. (B) Flow cytometry quantification of lipid accumulation. Unstained cells were used as a negative control (dotted line). Solid line represent MEFs stained with DODIPY, two independent experiments with similar results. (C) Before assay, WT MEFs were pretreated for 24hours with the indicated dose of Etomoxir. Realtime PCR analysis of Il-6 expression in MEFs stimulated with 100 ng/mL LPS for 3 hours. Data are expressed as the mean of duplicate reactions after normalization to expression of the gene encoding 18S rRNA and are representative of two independent experiments. [file mmc4.pdf]

**A***Il-6*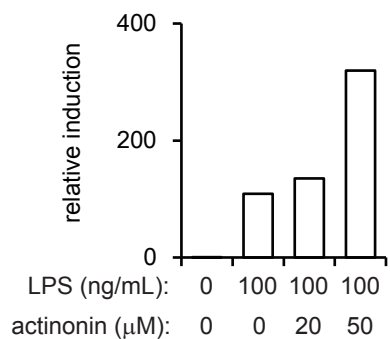**B***Il-6*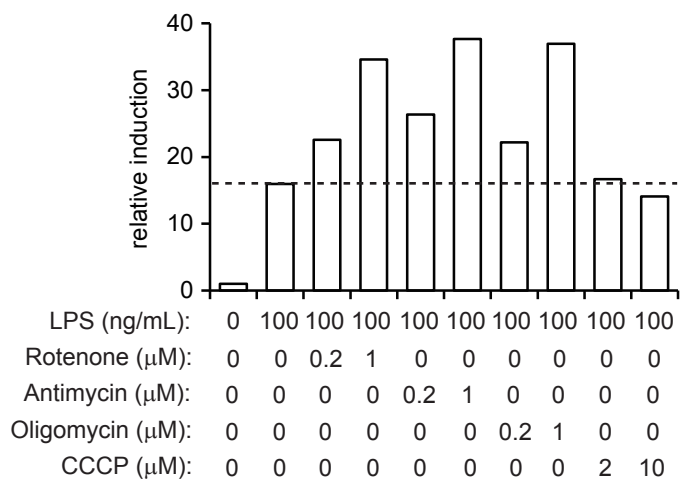

Supplement: Fig. S5 — Effect of actinonin on LPS − induced Il6 mRNA levels. (A) Before assay, WT MEFs were pretreated for 3 h with the indicated dose of actinonin. (B) Before assay, WT MEFs were pretreated for 3 h with the indicated dose of several mitochondrial inhibitors. (A,B) Real-time PCR analysis of Il6 expression in MEFs stimulated with 100 ng/mL LPS for 3 hours. Data are expressed as the mean of duplicate reactions after normalization to expression of the gene encoding 18S rRNA and are representative of two independent experiments. [file mmc5.pdf]
